# Symptoms and quality of life assessment after coil and foam embolization in patients with venous-origin chronic pelvic pain (VO-CPP) – a subgroup of pelvic venous disorders (PeVD)

**DOI:** 10.1080/07853890.2025.2570798

**Published:** 2025-10-11

**Authors:** Kamil Bałabuszek, Michał Toborek, Radosław Pietura

**Affiliations:** Department of Radiography, Medical University of Lublin, Lublin, Poland

**Keywords:** Venous-origin chronic pelvic pain, pelvic congestion syndrome, pelvic venous disorders, embolization, quality of life

## Abstract

**Background:**

Venous-Origin Chronic Pelvic Pain (VO-CPP), a subgroup of Pelvic Venous Disorders (PeVD) can significantly affect Quality of Life (QoL). Previous papers have highlighted the lack of evaluations measuring QoL of patients before and after embolization. The aim of this study was to evaluate the effectiveness of embolization in reducing a variety of symptoms and improving QoL in patients with VO-CPP.

**Methods:**

A prospective analysis of 40 female patients (mean 36.9 years) diagnosed with VO-CPP, undergoing venous embolization between June 2020 and May 2023 was conducted. Patients with extrinsic compressions, other pelvic conditions, and S_3_V_3_ without S_2_ in SVP Classification were excluded. Patients were evaluated before and after treatment at mid-term (MT) (9.4 ± 5.1 months) and long-term (LT) (29.6 ± 6.94 months). The severity of 12 different symptoms were assessed using VAS scale (pelvic, postcoital, standing, back, hip, leg and abdominal pain; nausea frequency, urinary discomfort, sleep disturbance and dysmenorrhoea), while QoL was measured using the SF-36 questionnaire. Patient satisfaction was assessed using the Likert scale.

**Results:**

Significant reductions in all measured symptoms were observed between pre-treatment and MT and LT follow-ups (*p* < 0.0001). No statistically significant differences were found between MT and LT scores, indicating a sustained relief. The greatest benefits were observed in reducing daytime pelvic pain (6.25 ± 1.93 to 2.49 ± 2.47), standing (7.43 ± 1.65 to 3.41 ± 2.43) and postcoital pain (6.40 ± 2.63 to 2.54 ± 2.22). QoL scores in both physical and mental health showed statistically significant and sustained improvement after the procedure. Most patients were satisfied with the procedure (81%), with 86% willing to undergo it again and 89% to recommend it.

**Conclusion:**

Embolization provides significant, sustained improvements in symptom relief while enhancing QoL in VO-CPP patients. Randomized controlled trials are needed to confirm these effects and exclude a placebo response.

## KEY MESSAGES

Embolization provides significant and sustained improvement in reducing a wide range of symptoms, and enhances the physical and mental health-related quality of life in patients with Venous-Origin Chronic Pelvic Pain (VO-CPP).

## Introduction

Pelvic Venous Disorders (PeVD), is a term for a chronic condition that affects a significant number of women worldwide. It results in a complex array of symptoms including chronic pelvic pain, development of varicose veins and significantly impacts patients’ quality of life. The pathophysiology results from venous insufficiency, where refluxing pelvic veins are responsible for the pain and other manifestations. Despite the prevalence of PeVD, it often remains underdiagnosed due to the broad spectrum of symptoms and the complexity of the condition [1]. Venous-Origin Chronic Pelvic Pain (VO-CPP) is a clinical presentation of PeVD, defined by pain symptoms perceived as originating from the pelvic organs, lasting longer than 6 months [2].

First described in 1993, endovascular embolization, a minimally invasive procedure targeting the affected veins, has emerged as a promising treatment option [3]. Studies report a significant decrease in pain levels, measured through the Visual Analog Scale (VAS), with 68.3% to 100% of patients experiencing marked relief after the procedure [4].

Despite the growing body of evidence supporting the efficacy of embolization, differences in procedural techniques, choice of embolic agents and patient selection criteria make it difficult to generalize results across studies [5]. Further research is still needed to validate these findings and to refine the technique [6]. The literature is still dominated by retrospective studies, and prospective analyses are limited, highlighting the importance of performing prospective studies [3].

Previous papers have highlighted the importance of assessing quality of life (QoL) in studies on this condition [3,7,8]. The study published by Kavallieros et al. found that QoL was the least frequently reported outcome [9]. This underreporting of QoL outcomes demonstrates the need for an approach that captures the broader impact of the disease and its treatment on patients’ lives [10].

This study addresses this gap by presenting prospective data on the effect of embolization in VO-CPP, using both VAS for detailed pain assessment and QoL analysis, to provide a holistic view of treatment impact. By focusing on these dual outcome measures, this study aims to contribute to the growing body of evidence supporting embolization as a viable treatment option for VO-CPP.

However, PeVD presents with a wide range of symptoms, from significant pelvic pain to pelvic-origin varicosities with minimal or no pain, leading to varied clinical presentations that can complicate outcome assessments. To ensure consistency in this study, we have concentrated on a specific subgroup of patients with VO-CPP – category S_2_V_2_ in the Symptoms-Varices-Patophysiology (SVP) Classification, previously referred to as Pelvic Congestion Syndrome, excluding those with varicose veins of pelvic origin who report minimal or no pain (S_3_V_3_ without S_2_) [11]. By focusing on patients with substantial pelvic pain, we aimed to provide a clear evaluation of embolization’s effectiveness in pain relief and its impact on related QOL outcomes, as these patients represent a subset where symptom alleviation is most urgently needed.

The primary objective of the study was to determine the efficacy of treatment in relation to various symptoms and QoL change, the secondary objective of the study was to determine whether daytime pelvic pain depends on the diameter of the ovarian veins and to assess patient satisfaction.

## Materials and methods

### Patients

This study was a prospective analysis of patients presenting to the Department of Interventional Radiology and Diagnostic Imaging with symptoms of VO-CPP between February 2020 and February 2023 who underwent coil and foam embolization. All patients had ultrasound and MRI or CT venography performed before the procedure, showing ovarian vein dilatation (>6 mm) and/or utero-vaginal plexus varices (>5 mm). The resulting data were assessed by two interventional radiologists to determine eligibility for embolization. Exclusion criteria included the presence of other pelvic pathologies (e.g. endometriosis), previous interventions involving the abdominal or pelvic veins, extrinsic iliac or renal compression, and hysterectomy. The study design was approved by the Bioethics Committee of the Medical University of Lublin (No. KE-0254/344/2019) and conducted in accordance with the principles of the Declaration of Helsinki. All included patients agreed to take part in the study and signed written informed consent.

Before the embolization procedure, patients were given two questionnaires:

- **Symptom Questionnaire:**
  - Patients were asked about the following 12 symptoms in the past 4 weeks: pelvic pain during the day and the night, pain during standing, dysmenorrhoea, dyspareunia, backache, hip pain, abdominal pain, urinary problems, leg pain, severity and frequency of nausea, sleep problems caused by pain.
  - The severity of each symptom was assessed using visual analogue scales (VAS). In this scale, patients rated their symptoms on a scale from 0 to 10, where 0 indicated the absence of pain, discomfort or symptoms and 10 indicated most severe pain, very frequent and severe discomfort or symptoms.
- **Quality of Life (QoL) Survey:**
  - The MOS 36-Item Short Form Survey Instrument (SF-36) developed by RAND as part of the Medical Outcomes Study.

Of the 82 enrolled patients, 15 were excluded from the study. Specifically, 8 patients were excluded because of the presence of other pelvic pathologies, 3 patients because of previous endovascular interventions involving the abdominal or pelvic veins or because of subsequent pelvic vein interventions performed at another institution during follow-up period. In addition, 1 patient withdrew consent, and 2 patients did not provide a valid contact e-mail address. One patient who also underwent a stenting procedure was excluded to provide uniformity of results.

**Figure 1.**
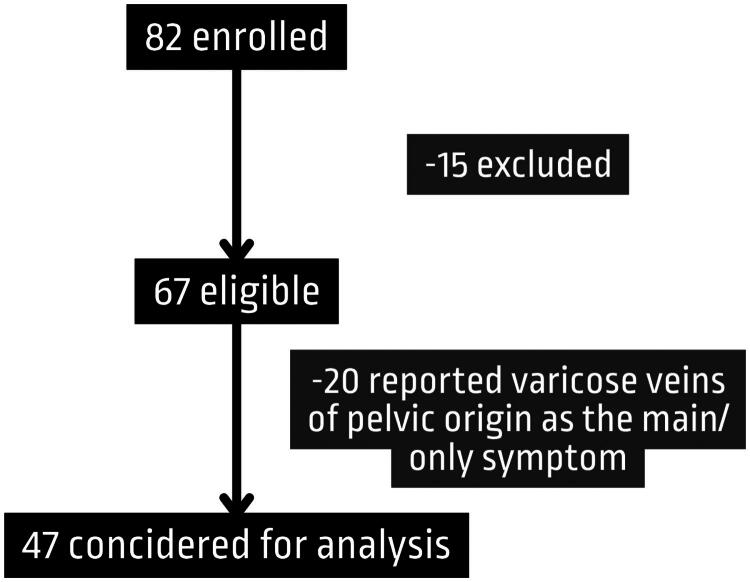
Study flow chart.

In this paper, we only analyzed patients who reported pelvic pain as their main symptom. Patients who reported only varicose veins of the pelvic origin without pain or varicose veins with mild pelvic or abdominal pain (< =4 VAS) were excluded from the analysis (20 patients). This left a final cohort of 47 patients eligible for the statistical analysis (Figure 1).

### Technique

The decision for the embolization target was made by the consensus of two operators on pre-procedure imaging and venography results, in which it was considered that the closure of the pelvic reflux could have an impact on clinical symptoms.

All embolization procedures were performed *via* the right jugular vein under ultrasound guidance using Seldinger’s technique with the local anaesthesia.

The 11 cm 7 Fr introducer sheath was inserted followed by a coaxial 90 cm 5 Fr introducer sheath. Then, using a 5 F catheter (Bentson, Merit Medical), a hydrophilic wire (Glidewire, Terumo) and a microcatheter 2.7Fr (Progreat, Terumo), a selective catheterization of the ovarian veins or branches of the internal iliac vein (IIV) was performed.

All procedures were performed using detachable coils, with the Ruby Coil (Penumbra) used in all 54 procedures in combination with the Interlock Coil (Boston Scientific) in 3 procedures. The most often used size was 8 mm × 60 cm.

After selective catheterization of the periuterine plexus, sclerotherapy was additionally performed by injecting a foam of 3% polidocanol (Aethoxysklerol, Kreussler Pharma) or tetradecyl sodium sulphate (Fibrovein, STD Pharmaceutical Products Ltd.). The foam was prepared using the Tessari technique with a 1:4 liquid-to-air ratio. The foam volume was administered based on the apparent size and flow characteristics of the venous plexus, with the upper limit up to 40 ml.

In the ovarian veins, the coils were placed in the lower segment of the vein trying to prevent obstruction of the deep pelvic venous plexus, while the upper part of the ovarian vein was left patent to maintain collateral drainage to the inferior vena cava, a crucial pathway in cases of extrinsic renal compression. In the uterine, internal pudendal and obturator veins coils have been placed at some distance from the main trunk of the IIV to avoid their migration. The size of the coil was chosen according to the calibre and morphology of the affected vein.

For patients presenting with extrapelvic varices in addition to pelvic/abdominal pain direct puncture of escape points (clitoral, glutaeal, inguinal, obturator, pudendal) for sclerotherapy was performed as needed. It was performed in a total of 8 patients. For managing complex cases, further embolizations, if necessary, were performed *via* jugular access. After the procedure, patients were kept in the hospital overnight for observation.

### Follow-up

Patients were sent two follow-up questionnaires *via* e-mail: **Symptom Questionnaire** using VAS and **QoL Assessment** using SF-36. Follow-up assessments were scheduled at 3, 6 and 12 months after the embolization procedure using the same follow-up questionnaires. However, not all participants responded at every of the three time points (only 11 patients). For the purposes of mid-term outcome analysis, we included each participant’s last available follow-up response to preserve sample size. Seven participants did not complete any of the follow-up questionnaires and were therefore excluded from the analysis.

**Figure 2.**
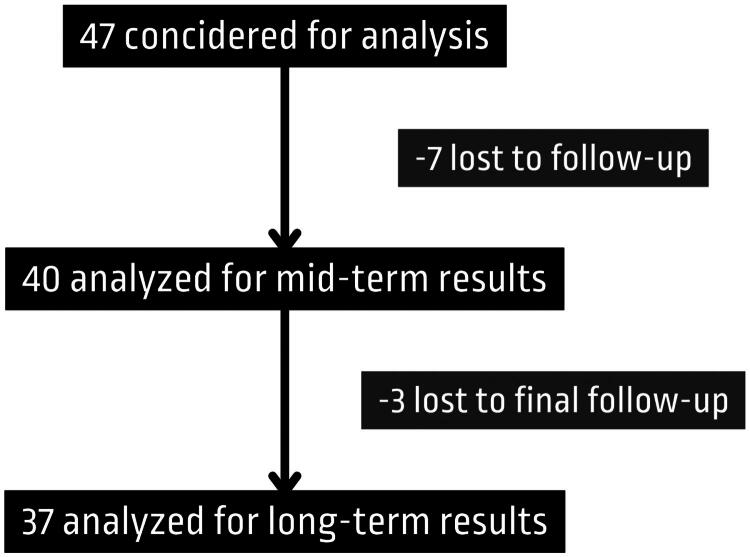
Post-intervention follow-up tracking.

Additionally to assess long-term outcomes of the procedure, a final follow-up was conducted *via* e-mail on 19 July 2024, with data collection occurring from 19 July to 18 August 2024. Multiple attempts were made to contact patients *via* phone and e-mail to maximize response rates. Three patients were lost to the final follow-up and did not provide any data (Figure 2).

During the final follow-up, patients were asked additional questions, including their overall satisfaction with the treatment, likelihood of recommending the procedure to a friend or choosing to undergo the procedure again, using a Likert scale. Patients were also asked whether they had undergone any additional interventions on pelvic veins at other facilities during the follow-up period.

### Statistical analysis

To achieve primary goals of the study, two measurement tools were utilized: the Symptom Questionnaire and the SF-36 questionnaire. Both tools were administered at three time points: before the procedure, after the intervention, and during the final follow-up.

The normality of the variables derived from the VAS scores on the Symptom Questionnaire was evaluated using the Shapiro-Wilk test. Since the data did not follow a normal distribution, non-parametric tests were applied. The Friedman test was conducted to assess differences in symptom severity across the three time points, followed by post-hoc Wilcoxon tests with Bonferroni correction to provide more detailed insights.

For the SF-36 questionnaire, responses were scored using the standard SF-36 algorithm, resulting in scores ranging from 0 to 100 for eight distinct health domains, where higher scores indicated better health outcomes. Based on literature, to facilitate interpretation these domains are commonly summarized into two components: the Physical Component Summary (PCS) and the Mental Component Summary (MCS) [12,13]. To determine whether a similar structure could be observed in our dataset, factor analysis with Varimax rotation was conducted independently for responses at each time point. Subsequently, differences in PCS and MCS scores across the three time points were evaluated using Repeated Measures ANOVA, with Tukey’s post-hoc tests applied to identify specific differences between time points. Parametric test assumptions were confirmed, with normality verified by the Shapiro-Wilk test.

Spearman’s correlation coefficient was calculated to test whether pelvic pain before the procedure was dependent on ovarian veins diameter, while patient satisfaction was measured using a Likert scale.

Statistical analyses and data visualization were performed using Python (version 3.10). The following libraries were employed: Pandas (1.5.3) for data preprocessing, SciPy (1.10.0) and Statsmodels (0.14.0) for statistical testing, and Matplotlib (3.7.0) and Seaborn (0.12.2) for visualizing the result.

## Results

### Patients

The analysis included 40 patients who underwent a total of 54 embolization procedures. Detailed demographic data are presented below (Table 1 and Table 2). Mid-term follow-up data were obtained from all 40 patients at a mean of 9.4 months (± 5.10), while long-term follow-up data were available for 37 patients at a mean of 29.6 months (± 6.94).

**Table 1.** Demographic data of patients.

	Mean	Median	SD	Min	Max
Age	36.9	35	8.58	21	63
Weight	61	62	9.59	44	80
Height	165.3	164	6.26	154	180
Pregnancies	2.1	2	1.57	0	7
Births	1.8	2	1.16	0	5

**Table 2.** Patients categorization in the symptoms-varices-patophysiology (SVP) classification.

Category	Number of patients (*n* = 40)
S_2_V_2_P_LG_V_,R,NT_	4
S_2,3b,_V_2,3b_P_BGV,R,NT;BIIV,R,NT;BPELV,R,NT_	4
S_2,3a,_V_2,3a_P_BGV,R,NT;BIIV,R,NT;BPELV,R,NT_	4
S_2_V_2_P_BGV,R,NT;LIIV,R,NT_	4
S_2_V_2_P_LGV,R,NT;LIIV,R,NT_	3
S_2_V_2_P_LGV,R,NT;BIIV,R,NT_	3
S_2_V_2_P_BGV,R,NT;BIIV,R,NT_	2
S_2,3b,_V_2,3b_P_BGV,R,NT;LIIV,R,NT;BPELV,R,NT_	2
S_2,3a,_V_2,3a_P_LGV,R,NT;BIIV,R,NT;BPELV,R,NT_	2
S_2_V_2_P_BGV,R,NT_	2
S_2,3b,_V_2,3b_P_LGV,R,NT;BIIV,R,NT;RPELV,R,NT_	1
S_2,3a,_V_2,3a_P_LGV,R,NT;BIIV,R,NT;LPELV,R,NT_	1
S_2,3b,_V_2,3b_P_RGV,R,NT;LIIV,R,NT;BPELV,R,NT_	1
S_2_V_2_P_BGV,R,NT;RIIV,R,NT_	1
S_2,3a,_V_2,3a_P_BGV,R,NT;RIIV,R,NT;BPELV,R,NT_	1
S_2_V_2_P_LIIV,R,NT_	1
S_2,3b,_V_2,3b_P_LGV,R,NT;BIIV,R,NT;BPELV,R,NT_	1
S_2,3a,_V_2,3a_P_BGV,R,NT;LPELV,R,NT_	1
S_2,3a,_V_2,3a_P_LGV,R,NT;LIIV,R,NT;BPELV,R,NT_	1
S_2,3b,_V_2,3b_P_LGV,R,NT;LPELV,R,NT_	1

A variety of venous combinations were treated. Highly selective embolization was performed on tributaries of the internal iliac vein, including left and right uterine veins, obturator veins, and pudendal veins. The most frequently treated vessel was the left ovarian vein (LOV), which was embolized in 95% of patients, followed by the left uterine vein in 75% of patients and the right ovarian vein (ROV) in 55% of patients. A detailed breakdown of treated veins is provided in the accompanying table (Table 3).

**Table 3.** Veins treated.

Vein	Percentage (%)
Left ovarian vein	95
Right ovarian vein	55
Left uterine vein	75
Right uterine vein	47.5
Left obturator vein	22.5
Right obturator vein	17.5
Left pudendal vein	32.5
Right pudendal vein	20

### VAS results

**Figure 3.**
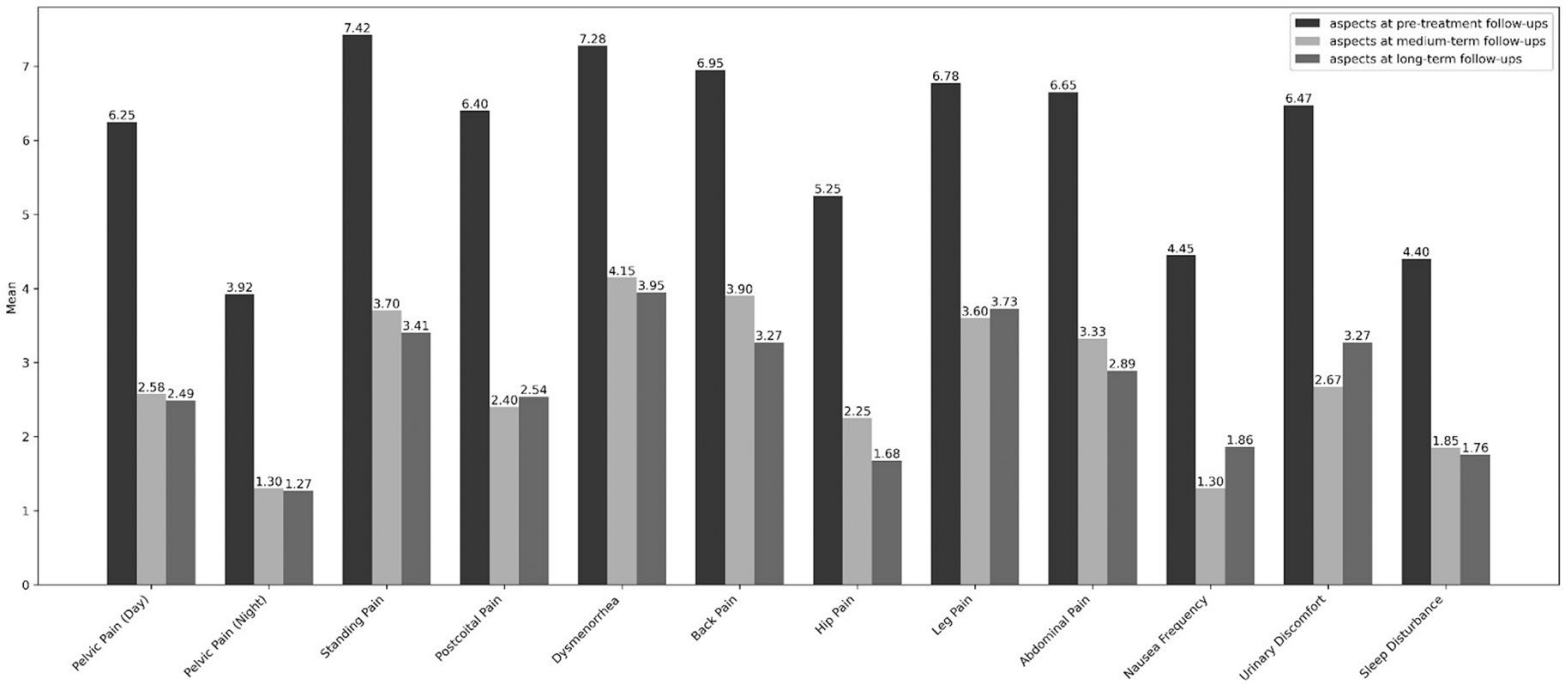
Change in visual analogue scale (VAS) pain scores before treatment, at mid- and long-term follow-up periods.

Overall, all analyzed symptoms showed significant reduction after treatment, indicating significant improvements across multiple symptom areas in both medium-term and long-term follow-ups. Friedman tests demonstrated a statistically significant reduction in pain levels when comparing pre-treatment scores with those at mid- and long-term follow-up. Post-hoc tests with Bonferroni correction confirmed that pain levels were significantly lower at both medium- and long-term follow-up compared to baseline (all *p* < 0.001). No significant differences were observed between medium-term and long-term follow-ups, with median pain scores fluctuating slightly but without statistical significance. Detailed results for all analyzed aspects at pre-treatment, medium-term, and long-term follow-ups are presented below (Table 4 and Figure 3).

**Table 4.** Results on the visual analogue scale (VAS) before and after treatment.

Variable	Pre-treatment	Medium-term	Long-term	Friedman test	Dunn-Bonferroni test (*p*-values)
Pelvic Pain (Day)	M ± SD: 6.25 (1.932) Median: 6	M ± SD: 2.58 (2.308) Median: 2	M ± SD: 2.49 (2.468) Median: 2	F: 42.88 *p*: ≪ 0.001	Pre-Medium: ≪ 0.001 Pre-Long: ≪ 0.001 Medium-Long: 1.292
Pelvic Pain (Night)	M ± SD: 3.93 (2.740) Median: 4	M ± SD: 1.30 (2.090) Median: 0	M ± SD: 1.27 (1.995) Median: 0	F: 37.48 *p*: ≪ 0.001	Pre-Medium:≪ 0.001 Pre-Long: ≪ 0.001 Medium-Long: 1.000
Standing Pain	M ± SD: 7.43 (1.647) Median: 7.5	M ± SD: 3.70 (2.691) Median: 3	M ± SD: 3.41 (2.432) Median: 3	F: 44.2 *p*: ≪ 0.001	Pre-Medium:≪ 0.001 Pre-Long: ≪ 0.001 Medium-Long: 1.000
Postcoital Pain	M ± SD: 6.40 (2.629) Median: 7	M ± SD: 2.40 (2.418) Median: 2	M ± SD: 2.54 (2.219) Median: 2	F: 35.17 *p*: ≪ 0.001	Pre-Medium: ≪ 0.001 Pre-Long: ≪ 0.001 Medium-Long: 1.000
Dysmenorrhoea	M ± SD: 7.28 (3.194) Median: 9	M ± SD: 4.15 (2.833) Median: 4	M ± SD: 3.95 (2.990) Median: 3	F: 30.83 *p*: ≪ 0.001	Pre-Medium: ≪ 0.001 Pre-Long: ≪ 0.001 Medium-Long: 0.816
Back Pain	M ± SD: 6.95 (2.253) Median: 7	M ± SD: 3.90 (2.570) Median: 3.5	M ± SD: 3.27 (2.589) Median: 3	F: 36.27 *p*: ≪ 0.001	Pre-Medium: ≪ 0.001 Pre-Long: ≪ 0.001 Medium-Long: 0.254
Hip Pain	M ± SD: 5.25 (2.976) Median: 6	M ± SD: 2.25 (2.478) Median: 2	M ± SD: 1.68 (2.028) Median: 1	F: 41.11 *p*: ≪ 0.001	Pre-Medium: ≪ 0.001 Pre-Long: ≪ 0.001 Medium-Long: 0.186
Leg Pain	M ± SD: 6.78 (2.567) Median: 8	M ± SD: 3.60 (2.697) Median: 3	M ± SD: 3.73 (2.785) Median: 3	F: 32.1 *p*: ≪ 0.001	Pre-Medium: ≪ 0.001 Pre-Long: ≪ 0.001 Medium-Long: 1.000
Abdominal Pain	M ± SD: 6.65 (2.167) Median: 7	M ± SD: 3.33 (2.390) Median: 3	M ± SD: 2.89 (2.590) Median: 2	F: 30.24 *p*: ≪ 0.001	Pre-Medium: ≪ 0.001 Pre-Long: ≪ 0.001 Medium-Long: 0.477
Nausea Frequency	M ± SD: 4.45 (3.021) Median: 5	M ± SD: 1.30 (1.488) Median: 1	M ± SD: 1.86 (2.371) Median: 1	F: 28.99 *p*: ≪ 0.001	Pre-Medium: ≪ 0.001 Pre-Long: ≪ 0.001 Medium-Long: 0.584
Urinary Discomfort	M ± SD: 6.48 (2.621) Median: 7	M ± SD: 2.68 (2.303) Median: 2	M ± SD: 3.27 (3.445) Median: 2	F: 35.8 *p*: ≪ 0.001	Pre-Medium: ≪ 0.001 Pre-Long: ≪ 0.001 Medium-Long: 1.000
Sleep Disturbance	M ± SD: 4.40 (3.319) Median: 5	M ± SD: 1.85 (2.568) Median: 0.5	M ± SD: 1.76 (2.543) Median: 1	F: 30.67 *p*: ≪ 0.001	Pre-Medium: ≪ 0.001 Pre-Long: ≪ 0.001 Medium-Long: 1.000

### SF-36 results

The SF-36 questionnaire results were analyzed using factor analysis with Varimax rotation. The two identified components corresponded to the Physical Component Summary (PCS) and the Mental Component Summary (MCS), consistent with existing literature [12,13] (Table 5).

**Table 5.** Summary of physical and mental health components and their related domains.

Component	Domains
Physical Component Summary (PCS)	*Physical Functioning, Role Physical, Bodily Pain, and General Health*
Mental Component Summary (MCS)	*Mental Health, Role Emotional, Social Functioning, and Vitality*

Repeated Measures ANOVA demonstrated significant differences for both components:

- For **PCS**, the test showed F = 14.90, *p* < 0.0001. Post-hoc Tukey tests confirmed that PCS scores were significantly lower before treatment compared to both medium-term and long-term follow-ups (*p* < 0.0001). No significant differences were observed between medium- and long-term measurements.
- For **MCS**, the test showed F = 7.97, *p* = 0.0007. Post-hoc Tukey tests revealed the same pattern as for PCS: significant improvements from pre-treatment to both medium- and long-term follow-ups (*p* < 0.0001), with no significant differences between medium-term and long-term results.

**Figure 4.**
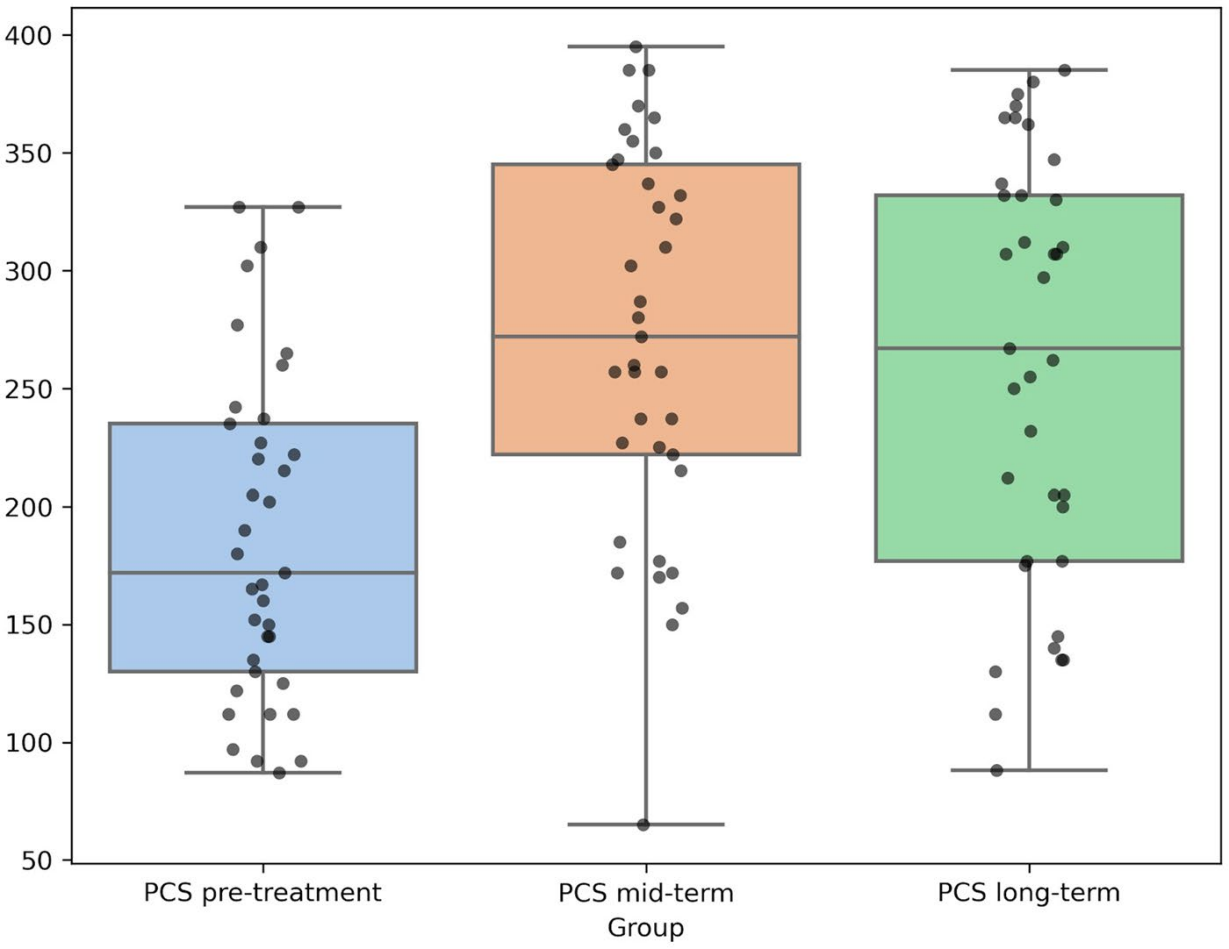
Box plots illustrating physical component summary (PCS) scores from the SF-36 questionnaire across three time points. Black dots represent individual patient scores. The coloured boxes represent the interquartile range (IQR), and the horizontal line inside each box indicates the median.

**Figure 5.**
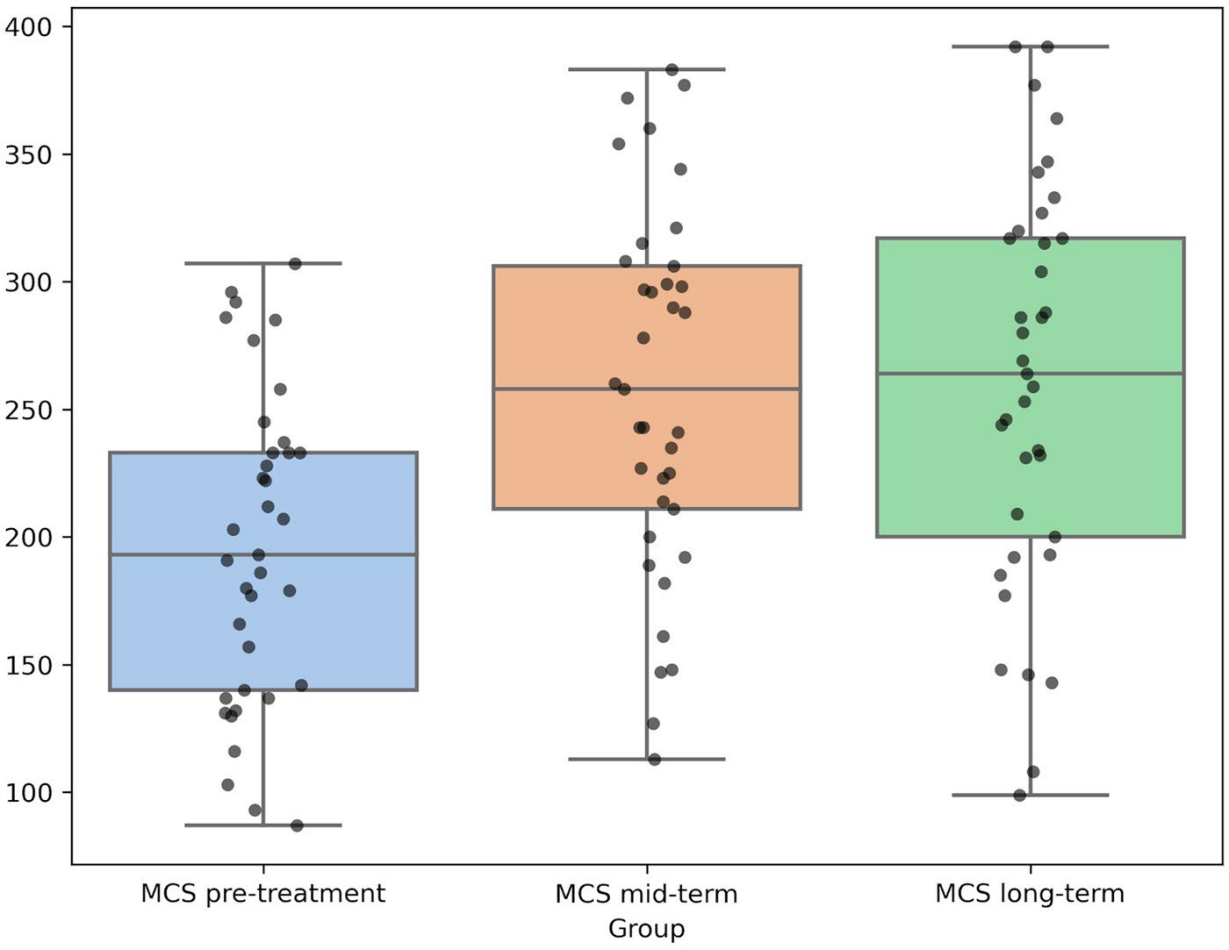
Box plots illustrating mental component summary (MCS) scores from the SF-36 questionnaire across three time points. Black dots represent individual patient scores. The coloured boxes represent the interquartile range (IQR), and the horizontal line inside each box indicates the median.

These results are visualized in Figures 4 and 5, using box plots to illustrate the distribution of PCS and MCS scores, highlighting the differences between groups (Figures 4 and 5). These results confirm that the intervention produced significant and sustained improvements in both physical health (PCS) and mental health (MCS), as well as pain levels in all measured parameters.

### Preprocedural measurements

Initial imaging studies (28 MRI, 12 CT) revealed the following gonadal vein diameters:

- The LOV diameter was 8.37 ± 2.17 mm, with a median of 8.8 mm (range: 3.1–13.0 mm).
- The ROV diameter was 6.16 ± 1.98 mm, with a median of 6.15 mm (range: 2.4–12.0 mm).

**Figure 6.**
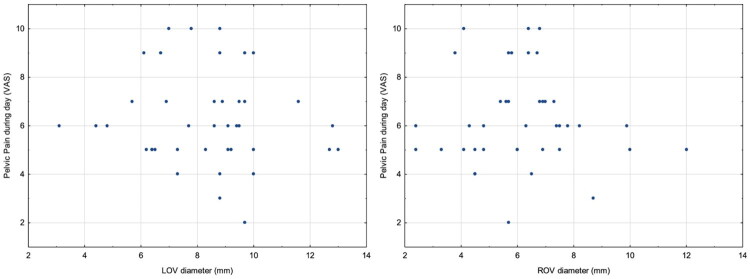
Scatter plot showing no correlation between reported daytime pelvic pain and gonadal vein diameters.

No relationship was observed between daytime pelvic pain and gonadal vein diameters. The lack of a statistically significant correlation was confirmed by Spearman correlation analysis, with a correlation coefficient of R=-0.13 (*p* = 0.43) for LOV and *R* = 0.003 (*p* = 0.98) for ROV (Figure 6).

### Patient satisfaction

**Figure 7.**
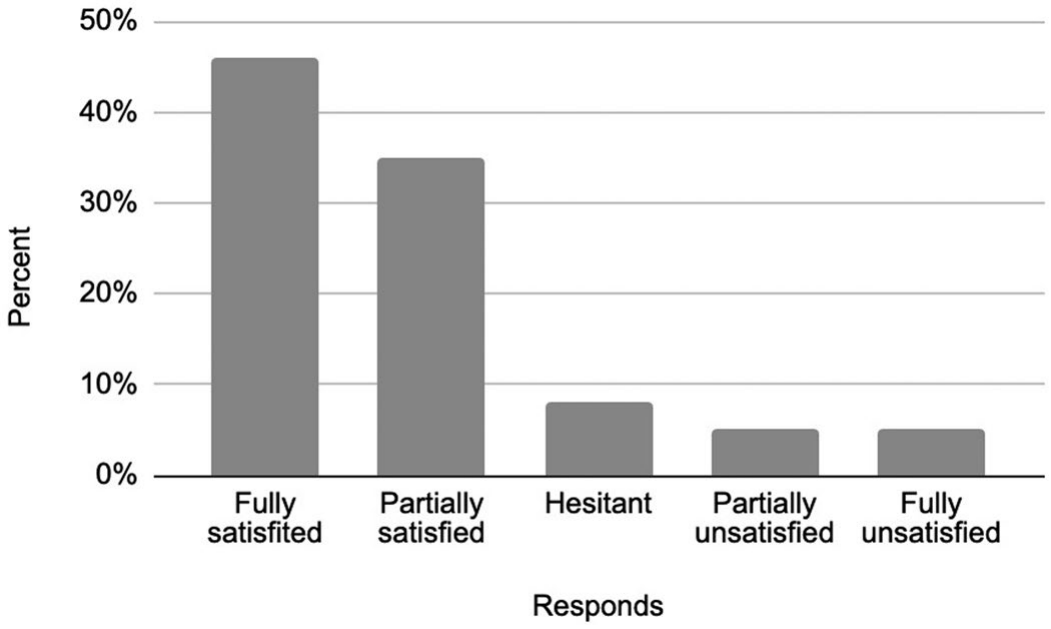
Overall satisfaction.

**Figure 8.**
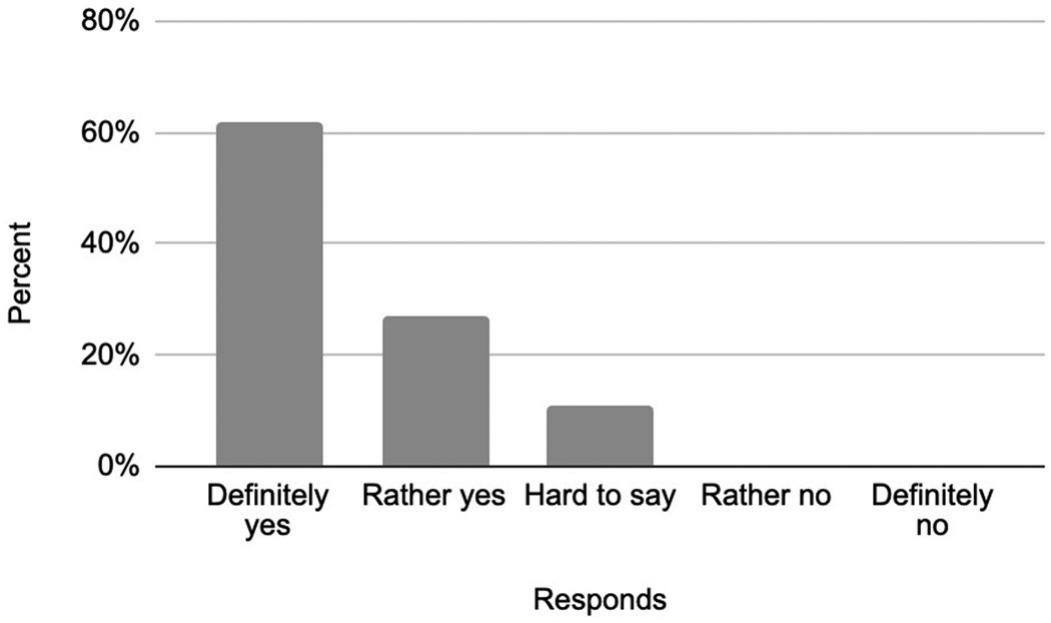
Likelihood of recommending the procedure.

**Figure 9.**
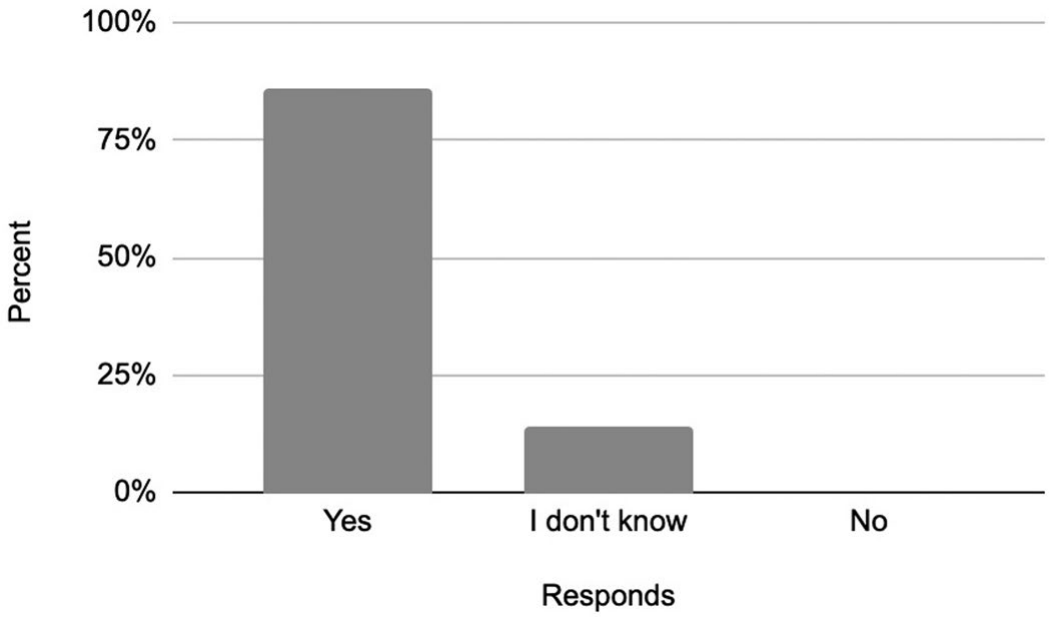
Responses to the question: ‘Would you choose to undergo the embolization procedure again?’

The satisfaction survey included three key aspects: overall satisfaction with the procedure, the likelihood of undergoing the procedure again, and the likelihood of recommending it to others. Results are summarized below (Figures 7–9).

These findings indicate that the majority of patients were satisfied with the procedure, would undergo it again, and would recommend it to others.

## Discussion

Embolization has been established as an effective modality for the treatment of VO-CPP for over three decades. However, the literature emphasizes the need for new evidence to sustain the strength of the recommendation for its use [6]. There is also a lack of papers using questionnaires to assess QoL before and after treatment [3,7–9,14].

The heterogeneity of PeVD symptoms, ranging from severe pelvic pain to asymptomatic varicose veins remains a challenge for research and clinical practice. Patients reporting significant pain cannot be directly compared to those with asymptomatic varices, as their clinical trajectories and treatment responses differ markedly [15]. Combining these groups in analyses risks diluting the observed effects of interventions. By excluding patients with minimal pain and focusing only on those with prominent symptoms, this study ensures a more homogeneous cohort. However, this approach may limit the generalisability of the results to the broader PeVD patient population.

This paper presents original data on the efficacy of coil and foam embolization in the treatment of various symptoms of VO-CPP by analysing pre- and postoperative outcomes across different symptoms. The findings of this study add to the growing body of evidence supporting the efficacy of embolization for managing symptoms connected with VO-CPP. While most previous studies have emphasized pelvic pain as the primary outcome, our evaluation extends the understanding of embolization’s impact by addressing multiple aspects of the condition [10,14]. By collecting detailed symptom-specific data, our research provides new perspectives on the manifestations of VO-CPP and the effectiveness of embolization.

Significant reductions in pain scores were observed across all measured parameters at both follow-ups when compared to pre-procedural levels. The absence of statistically significant differences between the mid-term (mean time 9.4 months) and long-term (mean time 29.6 months) evaluations indicates that the initial improvements observed after embolization were maintained over time.

Among the evaluated symptoms the greatest reductions were observed in pain during standing which decreased from a pre-treatment mean of 7.43 (±1.647) to 3.41 (±2.432), postcoital pain, which declined from 6.40 (±2.629) to 2.54 (±2.219), and the daytime pelvic pain which improved from 6.25 (±1.932) to 2.49 (±2.468). These findings suggest that embolization is particularly effective in addressing the venous insufficiency underlying these symptoms. Although all analyzed symptoms showed statistically significant improvements, night-time pelvic pain, sleep disturbances, and nausea frequency demonstrated less pronounced reductions. These symptoms are less commonly reported as typical for this condition and may arise from secondary factors or non-venous origins, suggesting that their response to embolization could be limited [16,17].

Similarly QoL, assessed *via* the SF-36 survey, demonstrated a significant improvement in both physical (PCS) and mental (MCS) well-being following the procedure. Similar to the trends observed in pain reduction, the improvements in PCS and MCS scores remained stable over time. No statistically significant differences were observed between mid-term and long-term follow-ups, suggesting that the initial improvements in QoL achieved after the procedure were maintained over time. This reinforces the importance of addressing not only the physical symptoms, but also the psychological burden of VO-CPP.

Patient-reported satisfaction further supports the clinical relevance of the intervention. A majority of participants reported being fully (46%) or partially satisfied (35%) with the procedure. These results are complemented by the findings on procedural acceptability, where 62% of patients indicated they would ‘definitely’ recommend the procedure to others, and an additional 27% responded ‘rather yes’. Similarly, 86% of participants stated they would undergo the procedure again. These satisfaction levels support the clinical utility of the intervention by its ability to address multiple dimensions of pain while garnering positive feedback from patients.

The analysis of the relationship between ovarian vein diameters and pelvic pain showed no significant correlation between these variables. Neither the left nor the right ovarian vein diameters influenced the severity of pelvic pain reported by patients before embolization. This lack of association was confirmed by Spearman correlation analysis, which showed no statistically significant relationship. These results suggest that patients with smaller ovarian veins can also experience significant pain, indicating that the presence and severity of symptoms may not directly depend on venous diameter alone. This result underscores the physiopathological complexity and multifactorial nature of pelvic pain in VO-CPP, emphasizing the need for a diagnostic approach that considers factors beyond anatomic measurements. These findings are consistent with existing evidence that ovarian vein diameter alone is an unreliable predictor of pain [17–21].

While most patients experienced sustained pelvic pain relief, a minority reported higher pain scores in the final questionnaire compared to the medium-term follow-up, suggesting a recurrence of symptoms. However, detailed face-to-face follow-up revealed that in some of these cases, the pain was localized in the vulvar varicose veins rather than the pelvic area typically associated with VO-CPP. This observation underscores the complexity of pelvic pain and underlines the need to categorize pelvic pain in the follow-ups more precisely. Distinguishing between internal and external venous sources of pelvic pain may be beneficial in future studies. These data align with the observed necessity for validated tools to categorize and assess symptoms more effectively, ensuring tailored and precise interventions [22].

One of the significant limitations of this study is the lack of a standardized protocol for selecting veins for embolization. Due to the complexity of the disease, the anatomical variability of the pelvic venous system, and the sources of reflux, the selection of target veins was based on phlebography and pre-procedural imaging studies. This variability underscores the ongoing debate in the literature, as there is no established consensus on the number or selection of veins that should be embolized to achieve optimal outcomes [4,7,23]. This discrepancy complicates comparisons between studies and underscores the need for evidence-based guidelines that define optimal procedural strategies for treating VO-CPP. Standardization of protocols across institutions could improve comparability of results and clarify the most effective approaches to treating VO-CPP.

A notable limitation of this study is the single-centre design and the lack of racial and ethnic diversity among respondents, which limits the generalisability of our findings. Additionally, our follow-up was based on electronic questionnaires. This approach was made because our patient cohort included individuals from across whole Poland, with many participants residing far from the treatment centre. After the procedure, many patients became less engaged in health-related communication. To obtain complementary data on long-term outcomes, we additionally conducted a final follow-up with extensive telephone and email reminders. While email-based questionnaires allow for facilitated data collection, they can lead to lower response rates and possible misinterpretation of sources of pain. This suggests that in-person follow-up, if feasible, may provide more accurate and consistent feedback.

Another limitation of the study is the relatively small number of participants. This was driven in large part by the COVID-19 pandemic, which lasted during the study period and significantly reduced hospital admissions for non-emergency treatments. Additionally, patients were assessed retrospectively using the SVP Classification, as it became available in 2021, during ongoing data collection of this study. It would be more beneficial if the study group was more homogeneous. Moreover, the prospective use of the SVP Classification would be more appropriate.

Although one of the strengths of this study is the inclusion of a QoL assessment, the evaluation was conducted using the SF-36 – a generic QoL measure. The absence of disease-specific instruments for patients with VO-CPP underscores the need for creating such a tool. Encouragingly, progress is being made in this area, with a patient-reported outcome measure currently under development [24]. In addition, the lack of information about patients’ use of other treatments and medications such as hormones or phlebotropic drugs during follow-up period may have affected the final results.

Lastly, the absence of very long-term follow-up data remains a common limitation in embolization studies for VO-CPP. While statistically significant improvements were observed over the study period, further investigation over multiple years is necessary to confirm the durability of these improvements. In addition, a key limitation of this and previous studies is the lack of a control group, which makes it impossible to draw definitive conclusions about the causal effect of the intervention, since the improvements observed after embolization may partly reflect a placebo effect. Randomized controlled trials with a sham control arm are desperately needed to provide high-quality evidence on treatment efficacy. The ongoing RCT in the USA is expected to offer important insights in this area [25].

## Conclusions

This study addressed a critical gap in VO-CPP research by incorporating detailed QoL assessments, measured using the SF-36 survey, which has been underreported in prior research. Our findings support embolization as a viable treatment method offering significant and sustained improvements in symptom relief, as well as enhancing both physical and mental health-related quality of life, providing a more holistic view of the intervention’s benefits. Despite its limitations, this study strengthens the evidence base for embolization as an effective treatment for VO-CPP. However, it also underscores the need for future research to address diagnostic challenges, procedural variability, and long-term follow-up strategies.

## Data Availability

The data that support the findings of this study are available from the corresponding author, K.B., upon reasonable request.

## References

[1] Bałabuszek K, Toborek M, Pietura R. Comprehensive overview of the venous disorder known as pelvic congestion syndrome. Ann Med. 2022;54(1):22–36. doi: 10.1080/07853890.2021.2014556. 34935563 PMC8725876

[2] Ford RW, Winokur RS. Pelvic venous disorders (PeVD). Semin Intervent Radiol. 2022;39(5):483–489. doi: 10.1055/s-0042-1757938. 36561941 PMC9767768

[3] Kashef E, Evans E, Patel N, et al. Pelvic venous congestion syndrome: female venous congestive syndromes and endovascular treatment options. CVIR Endovasc. 2023;6(1):25. doi: 10.1186/s42155-023-00365-y. 37076700 PMC10115924

[4] Brown CL, Rizer M, Alexander R, et al. Pelvic congestion syndrome: systematic review of treatment success. Semin Interv Radiol. 2018;35:35–40. 10.1055/s-0038-1636519 PMC5886772 29628614

[5] Tiralongo F, Distefano G, Palermo M, et al. Liquid and solid embolic agents in gonadal veins. J Clin Med. 2021;10(8):1596. doi: 10.3390/jcm10081596. 33918908 PMC8069975

[6] Champaneria R, Shah L, Moss J, et al. The relationship between pelvic vein incompetence and chronic pelvic pain in women: systematic reviews of diagnosis and treatment effectiveness. Health Technol Assess. 2016;20(5):1–108. doi: 10.3310/hta20050. PMC4781546 26789334

[7] Daniels JP, Champaneria R, Shah L, et al. Effectiveness of embolization or sclerotherapy of pelvic veins for reducing chronic pelvic pain: a systematic review. J Vasc Interv Radiol. 2016;27(10):1478–1486.e8. doi: 10.1016/j.jvir.2016.04.016. 27397619

[8] Clark MR, Taylor AC. Pelvic venous disorders: an update in terminology, diagnosis, and treatment. Semin Intervent Radiol. 2023;40(4):362–371. doi: 10.1055/s-0043-1771041. 37575340 PMC10415053

[9] Kavallieros K, Pope T, Tan M, et al. Identification of outcomes in clinical studies for pelvic venous disorders. J Vasc Surg Venous Lymphat Disord. 2024;12:101865. 38452895 10.1016/j.jvsv.2024.101865 PMC11523326

[10] Kavallieros K, Pope T, Mantonanakis K, et al. A scoping review of scores or grading systems for pelvic venous disorders. J Vasc Surg Venous Lymphat Disord. 2024;12(6):101901. doi: 10.1016/j.jvsv.2024.101901. 38677550 PMC11523442

[11] Meissner MH, Khilnani NM, Labropoulos N, et al. The symptoms-varices-pathophysiology classification of pelvic venous disorders: a report of the American Vein & Lymphatic Society International Working Group on pelvic venous disorders. J Vasc Surg Venous Lymphat Disord. 2021;9(3):568–584. doi: 10.1016/j.jvsv.2020.12.084. 33529720

[12] Fletcher BR, Damery S, Aiyegbusi OL, et al. Symptom burden and health-related quality of life in chronic kidney disease: a global systematic review and meta-analysis. PLoS Med. 2022;19(4):e1003954. doi: 10.1371/journal.pmed.1003954. 35385471 PMC8985967

[13] Ding X, Abner EL, Schmitt FA, et al. Mental component score (MCS) from health-related quality of life predicts incidence of dementia in U.S. males. J Prev Alzheimers Dis. 2021;8(2):169–174. doi: 10.14283/jpad.2020.50. 33569563 PMC8162937

[14] Hanna J, Bruinsma J, Temperley HC, et al. Efficacy of embolotherapy for the treatment of pelvic congestion syndrome: a systematic review. Ir J Med Sci. 2024;193(3):1441–1451. doi: 10.1007/s11845-024-03608-6. 38294607 PMC11128397

[15] Meissner MH, Gibson K. clinical outcome after treatment of pelvic congestion syndrome: sense and nonsense. Phlebology. 2015;30(1 Suppl):73–80. doi: 10.1177/0268355514568067. 25729071

[16] Patel SE, Chesnut SR. Relationships among pelvic congestion syndrome pain, daily activities, and quality of life. J Obstet Gynecol Neonatal Nurs. 2024;53(4):416–426. doi: 10.1016/j.jogn.2024.03.002. 38599242

[17] Antignani PL, Lazarashvili Z, Monedero JL, et al. Diagnosis and treatment of pelvic congestion syndrome: UIP consensus document. Int Angiol. 2019;38(4):265–283. doi: 10.23736/S0392-9590.19.04237-8. 31345010

[18] Dos Santos SJ, Holdstock JM, Harrison CC, et al. Ovarian vein diameter cannot be used as an indicator of ovarian venous reflux. Eur J Vasc Endovasc Surg. 2015;49(1):90–94. doi: 10.1016/j.ejvs.2014.10.013. 25457295

[19] Gavrilov S, Moskalenko YP, Mishakina NY, et al. Stratification of pelvic venous reflux in patients with pelvic varicose veins. J Vasc Surg Venous Lymphat Disord. 2021;9(6):1417–1424. doi: 10.1016/j.jvsv.2021.04.019. 34023538

[20] Jambon E, Le Bras Y, Petitpierre F, et al. MRI associated factors of clinical efficacy of embolization in patients with pelvic venous insufficiency. Diagn Interv Imaging. 2020;101(10):667–676. doi: 10.1016/j.diii.2020.06.004. 32713758

[21] Gavrilov S, Karalkin A, Mishakina N, et al. Relationships of pelvic vein diameter and reflux with clinical manifestations of pelvic venous disorder. Diagnostics. 2022;12(1):145. doi: 10.3390/diagnostics12010145. 35054312 PMC8774919

[22] Khilnani NM, Meissner MH, Learman LA, et al. Research priorities in pelvic venous disorders in women: recommendations from a multidisciplinary research consensus panel. J Vasc Interv Radiol. 2019;30(6):781–789. doi: 10.1016/j.jvir.2018.10.008. 30857986

[23] Mahmoud O, Vikatmaa P, Aho P, et al. Efficacy of endovascular treatment for pelvic congestion syndrome. J Vasc Surg Venous Lymphat Disord. 2016;4(3):355–370. doi: 10.1016/j.jvsv.2016.01.002. 27318059

[24] Khilnani NM. Development of a patient-reported outcome measure for women with chronic pelvic pain (PROM for CPP). Available online: https://clinicaltrials.gov/study/NCT06083597.

[25] Winokur RS. Trial of ovarian vein and pelvic vein embolization in women with chronic pelvic pain and pelvic varices (EMBOLIZE). Available online: https://clinicaltrials.gov/study/NCT06168058. 10.1016/j.jvir.2025.10.012 41110790

